# Bis(μ-3-carb­oxy-2-oxidobenzoato)-κ^3^O^1^,*O* ^2^:*O* ^3^;κ^3^ *O* ^3^:O^1^,*O* ^2^-bis­[aqua­(2,2′-bipyridine-κ^2^ *N*,*N*′)copper(II)]

**DOI:** 10.1107/S1600536808039913

**Published:** 2008-12-03

**Authors:** Jing Gao, Bao-Yong Zhu, De-Liang Cui

**Affiliations:** a Department of Chemistry and Chemical Engineering, Shandong University, Jinan 250100, People’s Republic of China; b Department of Chemistry, Dezhou University, Dezhou 253023, People’s Republic of China; c Institute of Crystalline Materials, Shandong University, Jinan 250100, People’s Republic of China

## Abstract

In the centrosymmetric dinuclear complex, [Cu_2_(C_8_H_4_O_5_)_2_(C_10_H_8_N_2_)_2_(H_2_O)_2_], the Cu^II^ ion is coordinated by two N atoms from a bipyridine ligand, three O atoms from two 3-carb­oxy-2-oxidobenzoate dianions and the O atom of the water mol­ecule in a distorted octa­hedral geometry. The Cu—-O(H) coordination [2.931 (3) Å] is very weak. In the crystal structure, the dinuclear units are linked into a two-dimensional network parallel to (010) by O—H⋯O hydrogen bonds.

## Related literature

For related structures, see: Augustin *et al.* (2005 ▶); Tao *et al.* (2002 ▶); Zheng *et al.* (2004 ▶).
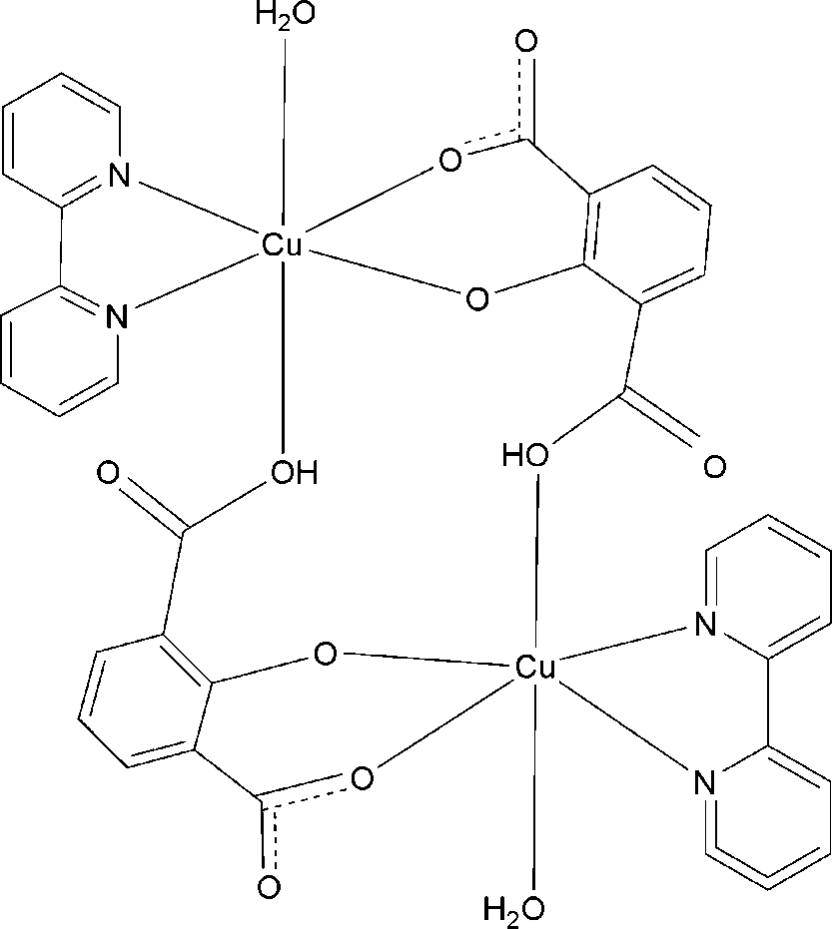


## Experimental

### 

#### Crystal data

[Cu_2_(C_8_H_4_O_5_)_2_(C_10_H_8_N_2_)_2_(H_2_O)_2_]
*M* *_r_* = 835.70
Triclinic, 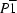

*a* = 8.354 (5) Å
*b* = 10.635 (5) Å
*c* = 11.038 (5) Å
α = 66.812 (5)°
β = 68.070 (5)°
γ = 89.269 (5)°
*V* = 825.8 (7) Å^3^
*Z* = 1
Mo *K*α radiation
μ = 1.36 mm^−1^
*T* = 293 (2) K
0.20 × 0.20 × 0.17 mm

#### Data collection

Bruker APEXII area-detector diffractometer
Absorption correction: multi-scan (*SADABS*; Bruker, 2005 ▶) *T*_min_ = 0.772, *T*_max_ = 0.801
4985 measured reflections
3686 independent reflections
2989 reflections with *I* > 2σ(*I*)
*R*_int_ = 0.016

#### Refinement

*R*[*F* ^2^ > 2σ(*F* ^2^)] = 0.035
*wR*(*F* ^2^) = 0.084
*S* = 1.17
3686 reflections
247 parameters
3 restraints
H-atom parameters constrained
Δρ_max_ = 0.32 e Å^−3^
Δρ_min_ = −0.25 e Å^−3^

### 

Data collection: *APEX2* (Bruker, 2005 ▶); cell refinement: *SAINT* (Bruker, 2005 ▶); data reduction: *SAINT*; program(s) used to solve structure: *SIR97* (Altomare *et al.*, 1999 ▶); program(s) used to refine structure: *SHELXL97* (Sheldrick, 2008 ▶); molecular graphics: *SHELXTL* (Sheldrick, 2008 ▶); software used to prepare material for publication: *WinGX* (Farrugia, 1999 ▶).

## Supplementary Material

Crystal structure: contains datablocks global, I. DOI: 10.1107/S1600536808039913/ci2713sup1.cif

Structure factors: contains datablocks I. DOI: 10.1107/S1600536808039913/ci2713Isup2.hkl

Additional supplementary materials:  crystallographic information; 3D view; checkCIF report

## Figures and Tables

**Table 1:** Selected bond lengths (Å)

Cu1—O3	1.8976 (18)
Cu1—O2	1.9325 (17)
Cu1—N2	2.004 (2)
Cu1—N1	2.007 (2)
Cu1—O1	2.301 (2)
Cu1—O5^i^	2.931 (2)

Symmetry code: (i) 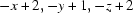.

**Table 2:** Hydrogen-bond geometry (Å, °)

*D*—H⋯*A*	*D*—H	H⋯*A*	*D*⋯*A*	*D*—H⋯*A*
O5—H5⋯O2	0.84	1.67	2.461 (2)	156
O1—H1*B*⋯O6^ii^	0.83	1.93	2.763 (3)	173
O1—H1*A*⋯O4^iii^	0.83	1.89	2.706 (3)	167

Symmetry codes: (ii) 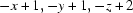; (iii) 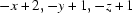.

**Fig. 1.**
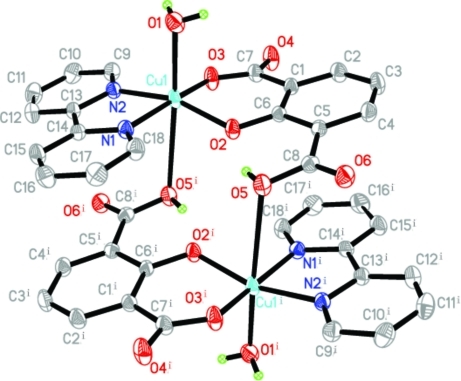
The structure of the title complex, showing the atom-labelling scheme. Displacement ellipsoids are drawn at the 50% probability level. Symmetry code: (i) -x + 2, -y + 1, -z + 2.

**Fig. 2.**
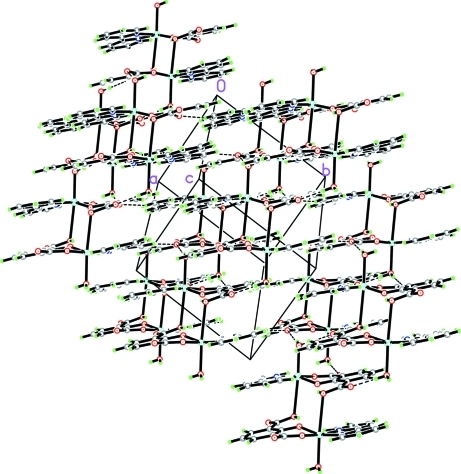
The crystal packing of the title compound. Hydrogen bonds are shown as dashed lines.

## supplementary crystallographic information

### Comment

2,2-Bipyridine and benzenedicarboxylate (BDC) anion are good organic ligands for copper which can construct supramolecular structure *via* hydrogen bonds and π-π aromatic interactions. We present here the crystal structure of [Cu_2_(bpy)_2_(ipO)_2_(H_2_O)_2_], where bpy is 2,2,-bipyridine and ipOH is 2-hydroxyisophthalate.

The title compound is a centrosymmetric dinuclear complex (Fig. 1). Each Cu^II^ ion is coordinated by two N atoms (N1 and N2) from a bipyridine ligand in a bidentate chelating fashion and three O atoms (O2, O3 and O5^i^) of two ipO dianions and the O atom (O1) of the solvent water molecule, in a distorted octahedral geometry. The Cu1—-O5^i^ coordination [2.931 (3) Å] is very weak. The Cu^II^ atom is located 0.209 (1) Å above the basal plane formed by atoms N1, N2, O2 and O3. The mean Cu—N(bpy) distance of 2.006 (2) Å and the bite angle N1—Cu1—N2 of 80.43 (8)° are close to the corresponding values observed in related copper-bipyridine compounds (Augustin *et al.*, 2005).

In the crystal structure, the centrosymmetric dinuclear units are linked into a two-dimensional network parallel to the (010) (Fig. 2) by O—H···O hydrogen bonds.

In [Cu_2_(bpy)_2_(ipO)_2_(H_2_O)_2_], the isophthalic acid (ipa) was *in situ* oxidative hydroxylated before coordinating with Cu^II^ ion (Tao *et al.*, 2002). Similar *in situ* oxidation has also been reported for 1,2,3-benzenetricarboxylic acid (Zheng *et al.*, 2004).

### Experimental

Copper(II) chloride dihydrate (0.043 g, 0.251 mol), 2,2,-bipyridine (0.039 g, 0.249 mol), isophthalic acid (0.083 g, 0.500 mol), potassium hydroxide (0.055 g, 0.982 mol) and deionized water (18 ml) were mixed together. The mixture was sealed in a Teflon-lined stainless steel vessel (25 ml) and then heated at 453 K for 36 h under autogenous pressure and then cooled to room temperature. Light-green crystals were obtained by slow evaporation of the mother-liquor in the air for a few days.

### Refinement

O-bound H atoms were located in a difference map and then restrained to ride on their parent atoms, with a O-H distance of 0.84 (1) Å. C-bound H atoms were positioned geometrically and treated as riding, with C-H = 0.93 Å and *U*_iso_(H) = 1.2*U*_eq_(C).

### Crystal data

**Table:**

[Cu_2_(C_8_H_4_O_5_)_2_(C_10_H_8_N_2_)_2_(H_2_O)_2_]	*Z* = 1
*M**_r_* = 835.70	*F*(000) = 426
Triclinic, *P*1	*D*_x_ = 1.680 Mg m^−^^3^
Hall symbol: -P 1	Mo *K*α radiation, λ = 0.71073 Å
*a* = 8.354 (5) Å	Cell parameters from 3943 reflections
*b* = 10.635 (5) Å	θ = 2.1–28.2°
*c* = 11.038 (5) Å	µ = 1.36 mm^−^^1^
α = 66.812 (5)°	*T* = 293 K
β = 68.070 (5)°	Block, green
γ = 89.269 (5)°	0.20 × 0.20 × 0.17 mm
*V* = 825.8 (7) Å^3^	

### Data collection

**Table:**

Bruker APEXII area-detector diffractometer	3686 independent reflections
Radiation source: fine-focus sealed tube	2989 reflections with *I* > 2σ(*I*)
graphite	*R*_int_ = 0.016
φ and ω scans	θ_max_ = 28.2°, θ_min_ = 2.1°
Absorption correction: multi-scan (*SADABS*; Bruker, 2005)	*h* = −11→10
*T*_min_ = 0.772, *T*_max_ = 0.801	*k* = −11→14
4985 measured reflections	*l* = −14→14

### Refinement

**Table:**

Refinement on *F*^2^	Primary atom site location: structure-invariant direct methods
Least-squares matrix: full	Secondary atom site location: difference Fourier map
*R*[*F*^2^ > 2σ(*F*^2^)] = 0.036	Hydrogen site location: inferred from neighbouring sites
*wR*(*F*^2^) = 0.084	H-atom parameters constrained
*S* = 1.17	*w* = 1/[σ^2^(*F*_o_^2^) + (0.0284*P*)^2^ + 0.2*P*] where *P* = (*F*_o_^2^ + 2*F*_c_^2^)/3
3686 reflections	(Δ/σ)_max_ = 0.001
247 parameters	Δρ_max_ = 0.32 e Å^−^^3^
3 restraints	Δρ_min_ = −0.25 e Å^−^^3^

### Special details

**Table:**

Geometry. All e.s.d.'s (except the e.s.d. in the dihedral angle between two l.s. planes) are estimated using the full covariance matrix. The cell e.s.d.'s are taken into account individually in the estimation of e.s.d.'s in distances, angles and torsion angles; correlations between e.s.d.'s in cell parameters are only used when they are defined by crystal symmetry. An approximate (isotropic) treatment of cell e.s.d.'s is used for estimating e.s.d.'s involving l.s. planes.
Refinement. Refinement of *F*^2^ against ALL reflections. The weighted *R*-factor *wR* and goodness of fit *S* are based on *F*^2^, conventional *R*-factors *R* are based on *F*, with *F* set to zero for negative *F*^2^. The threshold expression of *F*^2^ > σ(*F*^2^) is used only for calculating *R*-factors(gt) *etc*. and is not relevant to the choice of reflections for refinement. *R*-factors based on *F*^2^ are statistically about twice as large as those based on *F*, and *R*- factors based on ALL data will be even larger.

### Fractional atomic coordinates and isotropic or equivalent isotropic displacement parameters (Å^2^)

**Table:**

	*x*	*y*	*z*	*U*_iso_*/*U*_eq_	
Cu1	1.02424 (4)	0.40419 (3)	0.79285 (3)	0.03492 (11)	
O1	0.8041 (2)	0.2876 (2)	0.78123 (19)	0.0477 (5)	
H1A	0.8460	0.2870	0.7003	0.050 (9)*	
H1B	0.7220	0.3329	0.7808	0.073 (11)*	
O2	0.8826 (2)	0.50132 (17)	0.89930 (16)	0.0386 (4)	
O5	0.7156 (2)	0.47764 (18)	1.14597 (18)	0.0428 (4)	
H5	0.7925	0.4761	1.0720	0.079 (11)*	
O3	1.0772 (2)	0.56037 (18)	0.61539 (18)	0.0475 (5)	
O6	0.4835 (2)	0.5775 (2)	1.19418 (19)	0.0472 (5)	
O4	1.0474 (3)	0.7547 (2)	0.46051 (18)	0.0546 (5)	
C1	0.8480 (3)	0.6833 (2)	0.6998 (2)	0.0328 (5)	
C2	0.7521 (3)	0.7865 (3)	0.6566 (3)	0.0429 (6)	
H2	0.7839	0.8396	0.5588	0.052*	
N2	1.2225 (3)	0.3176 (2)	0.7039 (2)	0.0342 (5)	
C5	0.6589 (3)	0.6344 (2)	0.9478 (2)	0.0319 (5)	
C8	0.6095 (3)	0.5615 (3)	1.1045 (3)	0.0351 (5)	
C6	0.8000 (3)	0.6034 (2)	0.8478 (2)	0.0297 (5)	
C14	1.1417 (3)	0.1660 (2)	0.9492 (3)	0.0344 (5)	
N1	1.0135 (3)	0.2428 (2)	0.9707 (2)	0.0343 (5)	
C13	1.2642 (3)	0.2115 (2)	0.7976 (3)	0.0345 (5)	
C9	1.3259 (3)	0.3668 (3)	0.5628 (3)	0.0420 (6)	
H9	1.2961	0.4392	0.4976	0.050*	
C10	1.4742 (4)	0.3132 (3)	0.5122 (3)	0.0507 (7)	
H10	1.5434	0.3484	0.4142	0.061*	
C4	0.5662 (3)	0.7384 (3)	0.8979 (3)	0.0416 (6)	
H4	0.4723	0.7571	0.9635	0.050*	
C7	0.9998 (3)	0.6649 (3)	0.5847 (3)	0.0371 (6)	
C3	0.6113 (4)	0.8133 (3)	0.7535 (3)	0.0482 (7)	
H3	0.5477	0.8815	0.7212	0.058*	
C12	1.4127 (4)	0.1550 (3)	0.7521 (3)	0.0485 (7)	
H12	1.4408	0.0823	0.8182	0.058*	
C11	1.5186 (4)	0.2078 (3)	0.6077 (3)	0.0536 (8)	
H11	1.6199	0.1717	0.5755	0.064*	
C15	1.1534 (4)	0.0535 (3)	1.0627 (3)	0.0464 (7)	
H15	1.2411	0.0001	1.0461	0.056*	
C17	0.9043 (4)	0.1000 (3)	1.2207 (3)	0.0521 (7)	
H17	0.8221	0.0799	1.3130	0.063*	
C18	0.8958 (4)	0.2081 (3)	1.1050 (3)	0.0442 (6)	
H18	0.8051	0.2593	1.1202	0.053*	
C16	1.0337 (4)	0.0218 (3)	1.2004 (3)	0.0537 (8)	
H16	1.0410	−0.0519	1.2783	0.064*	

### Atomic displacement parameters (Å^2^)

**Table:**

	*U*^11^	*U*^22^	*U*^33^	*U*^12^	*U*^13^	*U*^23^
Cu1	0.04080 (18)	0.03336 (18)	0.02357 (16)	0.01615 (13)	−0.01105 (12)	−0.00717 (12)
O1	0.0447 (10)	0.0642 (13)	0.0384 (11)	0.0230 (10)	−0.0178 (9)	−0.0249 (10)
O2	0.0488 (10)	0.0383 (10)	0.0242 (8)	0.0205 (8)	−0.0135 (7)	−0.0103 (7)
O5	0.0467 (10)	0.0488 (11)	0.0264 (9)	0.0202 (9)	−0.0107 (8)	−0.0137 (8)
O3	0.0555 (11)	0.0443 (11)	0.0262 (9)	0.0261 (9)	−0.0092 (8)	−0.0059 (8)
O6	0.0440 (10)	0.0587 (12)	0.0378 (10)	0.0187 (9)	−0.0114 (8)	−0.0241 (9)
O4	0.0678 (13)	0.0475 (11)	0.0248 (9)	0.0273 (10)	−0.0096 (9)	−0.0011 (8)
C1	0.0371 (13)	0.0301 (13)	0.0296 (12)	0.0095 (10)	−0.0145 (10)	−0.0100 (10)
C2	0.0507 (16)	0.0395 (15)	0.0331 (14)	0.0143 (12)	−0.0185 (12)	−0.0085 (12)
N2	0.0383 (11)	0.0321 (11)	0.0316 (11)	0.0103 (9)	−0.0150 (9)	−0.0120 (9)
C5	0.0338 (12)	0.0318 (13)	0.0309 (12)	0.0079 (10)	−0.0134 (10)	−0.0136 (10)
C8	0.0346 (13)	0.0369 (14)	0.0344 (13)	0.0052 (11)	−0.0119 (11)	−0.0175 (11)
C6	0.0330 (12)	0.0277 (12)	0.0288 (12)	0.0082 (10)	−0.0136 (10)	−0.0110 (10)
C14	0.0427 (14)	0.0268 (12)	0.0368 (13)	0.0081 (10)	−0.0214 (11)	−0.0112 (11)
N1	0.0406 (11)	0.0293 (11)	0.0287 (10)	0.0083 (9)	−0.0135 (9)	−0.0083 (9)
C13	0.0378 (13)	0.0291 (13)	0.0386 (14)	0.0085 (10)	−0.0180 (11)	−0.0136 (11)
C9	0.0462 (15)	0.0398 (15)	0.0351 (14)	0.0097 (12)	−0.0124 (12)	−0.0148 (12)
C10	0.0457 (16)	0.0519 (18)	0.0434 (16)	0.0078 (13)	−0.0042 (13)	−0.0222 (14)
C4	0.0396 (14)	0.0444 (15)	0.0417 (15)	0.0171 (12)	−0.0144 (12)	−0.0210 (12)
C7	0.0430 (14)	0.0350 (14)	0.0262 (12)	0.0098 (11)	−0.0135 (11)	−0.0064 (11)
C3	0.0534 (16)	0.0428 (16)	0.0479 (16)	0.0251 (13)	−0.0246 (14)	−0.0149 (13)
C12	0.0488 (16)	0.0416 (16)	0.0586 (18)	0.0202 (13)	−0.0248 (14)	−0.0216 (14)
C11	0.0397 (15)	0.0534 (18)	0.062 (2)	0.0163 (13)	−0.0105 (14)	−0.0286 (16)
C15	0.0559 (17)	0.0329 (14)	0.0512 (17)	0.0139 (12)	−0.0295 (14)	−0.0108 (13)
C17	0.0705 (19)	0.0396 (16)	0.0288 (14)	0.0033 (14)	−0.0141 (13)	−0.0027 (12)
C18	0.0507 (16)	0.0360 (14)	0.0351 (14)	0.0103 (12)	−0.0132 (12)	−0.0082 (12)
C16	0.076 (2)	0.0358 (15)	0.0418 (16)	0.0098 (14)	−0.0311 (15)	−0.0020 (13)

### Geometric parameters (Å, °)

**Table:**

Cu1—O3	1.8976 (18)	C5—C8	1.477 (3)
Cu1—O2	1.9325 (17)	C14—N1	1.346 (3)
Cu1—N2	2.004 (2)	C14—C15	1.385 (3)
Cu1—N1	2.007 (2)	C14—C13	1.475 (3)
Cu1—O1	2.301 (2)	N1—C18	1.338 (3)
Cu1—O5^i^	2.931 (2)	C13—C12	1.380 (3)
O1—H1A	0.83	C9—C10	1.374 (4)
O1—H1B	0.83	C9—H9	0.93
O2—C6	1.328 (3)	C10—C11	1.362 (4)
O5—C8	1.324 (3)	C10—H10	0.93
O5—H5	0.84	C4—C3	1.369 (4)
O3—C7	1.272 (3)	C4—H4	0.93
O6—C8	1.211 (3)	C3—H3	0.93
O4—C7	1.233 (3)	C12—C11	1.376 (4)
C1—C2	1.387 (3)	C12—H12	0.93
C1—C6	1.407 (3)	C11—H11	0.93
C1—C7	1.499 (3)	C15—C16	1.376 (4)
C2—C3	1.378 (4)	C15—H15	0.93
C2—H2	0.93	C17—C16	1.361 (4)
N2—C13	1.344 (3)	C17—C18	1.367 (4)
N2—C9	1.346 (3)	C17—H17	0.93
C5—C4	1.394 (3)	C18—H18	0.93
C5—C6	1.422 (3)	C16—H16	0.93
			
O3—Cu1—O2	91.52 (8)	C15—C14—C13	124.1 (2)
O3—Cu1—N2	92.25 (8)	C18—N1—C14	118.6 (2)
O2—Cu1—N2	162.60 (8)	C18—N1—Cu1	126.46 (17)
O3—Cu1—N1	169.99 (8)	C14—N1—Cu1	114.96 (15)
O2—Cu1—N1	93.53 (8)	N2—C13—C12	121.3 (2)
N2—Cu1—N1	80.43 (8)	N2—C13—C14	114.3 (2)
O3—Cu1—O1	96.20 (8)	C12—C13—C14	124.5 (2)
O2—Cu1—O1	98.23 (8)	N2—C9—C10	121.9 (3)
N2—Cu1—O1	98.25 (8)	N2—C9—H9	119.0
N1—Cu1—O1	91.63 (8)	C10—C9—H9	119.0
O3—Cu1—O5^i^	92.34 (8)	C11—C10—C9	119.1 (3)
O2—Cu1—O5^i^	79.41 (8)	C11—C10—H10	120.5
N2—Cu1—O5^i^	83.47 (8)	C9—C10—H10	120.5
N1—Cu1—O5^i^	80.11 (8)	C3—C4—C5	120.9 (2)
O1—Cu1—O5^i^	171.22 (6)	C3—C4—H4	119.5
Cu1—O1—H1A	105.3	C5—C4—H4	119.5
Cu1—O1—H1B	110.7	O4—C7—O3	121.6 (2)
H1A—O1—H1B	104.9	O4—C7—C1	117.8 (2)
C6—O2—Cu1	124.77 (15)	O3—C7—C1	120.6 (2)
C8—O5—H5	107.2	C4—C3—C2	119.3 (2)
C7—O3—Cu1	130.12 (16)	C4—C3—H3	120.3
C2—C1—C6	118.8 (2)	C2—C3—H3	120.3
C2—C1—C7	117.6 (2)	C11—C12—C13	119.1 (3)
C6—C1—C7	123.6 (2)	C11—C12—H12	120.4
C3—C2—C1	122.4 (2)	C13—C12—H12	120.4
C3—C2—H2	118.8	C10—C11—C12	119.7 (3)
C1—C2—H2	118.8	C10—C11—H11	120.2
C13—N2—C9	118.9 (2)	C12—C11—H11	120.2
C13—N2—Cu1	115.19 (16)	C16—C15—C14	119.2 (3)
C9—N2—Cu1	125.39 (17)	C16—C15—H15	120.4
C4—C5—C6	119.7 (2)	C14—C15—H15	120.4
C4—C5—C8	118.5 (2)	C16—C17—C18	119.8 (3)
C6—C5—C8	121.8 (2)	C16—C17—H17	120.1
O6—C8—O5	119.4 (2)	C18—C17—H17	120.1
O6—C8—C5	124.2 (2)	N1—C18—C17	122.2 (3)
O5—C8—C5	116.3 (2)	N1—C18—H18	118.9
O2—C6—C1	123.1 (2)	C17—C18—H18	118.9
O2—C6—C5	118.1 (2)	C17—C16—C15	118.9 (3)
C1—C6—C5	118.8 (2)	C17—C16—H16	120.5
N1—C14—C15	121.3 (2)	C15—C16—H16	120.5
N1—C14—C13	114.6 (2)		
			
O3—Cu1—O2—C6	27.15 (19)	O2—Cu1—N1—C14	−158.90 (17)
N2—Cu1—O2—C6	129.6 (3)	N2—Cu1—N1—C14	4.66 (17)
N1—Cu1—O2—C6	−161.50 (19)	O1—Cu1—N1—C14	102.75 (18)
O1—Cu1—O2—C6	−69.34 (19)	C9—N2—C13—C12	1.7 (4)
O2—Cu1—O3—C7	−19.2 (2)	Cu1—N2—C13—C12	−170.8 (2)
N2—Cu1—O3—C7	177.8 (2)	C9—N2—C13—C14	−179.9 (2)
N1—Cu1—O3—C7	−139.5 (4)	Cu1—N2—C13—C14	7.6 (3)
O1—Cu1—O3—C7	79.3 (2)	N1—C14—C13—N2	−3.7 (3)
C6—C1—C2—C3	−0.2 (4)	C15—C14—C13—N2	176.0 (2)
C7—C1—C2—C3	−179.3 (2)	N1—C14—C13—C12	174.7 (2)
O3—Cu1—N2—C13	166.35 (18)	C15—C14—C13—C12	−5.6 (4)
O2—Cu1—N2—C13	64.0 (3)	C13—N2—C9—C10	−1.1 (4)
N1—Cu1—N2—C13	−6.78 (17)	Cu1—N2—C9—C10	170.6 (2)
O1—Cu1—N2—C13	−97.06 (18)	N2—C9—C10—C11	−0.5 (4)
O3—Cu1—N2—C9	−5.6 (2)	C6—C5—C4—C3	−1.4 (4)
O2—Cu1—N2—C9	−107.9 (3)	C8—C5—C4—C3	177.2 (2)
N1—Cu1—N2—C9	−178.7 (2)	Cu1—O3—C7—O4	−176.2 (2)
O1—Cu1—N2—C9	91.0 (2)	Cu1—O3—C7—C1	4.4 (4)
C4—C5—C8—O6	6.7 (4)	C2—C1—C7—O4	10.8 (4)
C6—C5—C8—O6	−174.7 (2)	C6—C1—C7—O4	−168.3 (2)
C4—C5—C8—O5	−170.9 (2)	C2—C1—C7—O3	−169.8 (3)
C6—C5—C8—O5	7.6 (3)	C6—C1—C7—O3	11.1 (4)
Cu1—O2—C6—C1	−21.3 (3)	C5—C4—C3—C2	−0.9 (4)
Cu1—O2—C6—C5	160.03 (16)	C1—C2—C3—C4	1.8 (4)
C2—C1—C6—O2	179.2 (2)	N2—C13—C12—C11	−0.7 (4)
C7—C1—C6—O2	−1.8 (4)	C14—C13—C12—C11	−178.9 (3)
C2—C1—C6—C5	−2.2 (4)	C9—C10—C11—C12	1.4 (5)
C7—C1—C6—C5	176.8 (2)	C13—C12—C11—C10	−0.8 (4)
C4—C5—C6—O2	−178.3 (2)	N1—C14—C15—C16	−1.5 (4)
C8—C5—C6—O2	3.1 (3)	C13—C14—C15—C16	178.8 (2)
C4—C5—C6—C1	3.0 (4)	C14—N1—C18—C17	1.7 (4)
C8—C5—C6—C1	−175.6 (2)	Cu1—N1—C18—C17	−176.6 (2)
C15—C14—N1—C18	−0.2 (4)	C16—C17—C18—N1	−1.5 (5)
C13—C14—N1—C18	179.5 (2)	C18—C17—C16—C15	−0.2 (5)
C15—C14—N1—Cu1	178.29 (19)	C14—C15—C16—C17	1.6 (4)
C13—C14—N1—Cu1	−2.0 (3)	O5^i^—Cu1—O2—C6	119.26 (19)
O3—Cu1—N1—C18	139.6 (4)	O5^i^—Cu1—O3—C7	−98.6 (3)
O2—Cu1—N1—C18	19.4 (2)	O5^i^—Cu1—N2—C13	74.20 (18)
N2—Cu1—N1—C18	−177.0 (2)	O5^i^—Cu1—N2—C9	−97.7 (2)
O1—Cu1—N1—C18	−78.9 (2)	O5^i^—Cu1—N1—C18	98.1 (2)
O3—Cu1—N1—C14	−38.7 (5)	O5^i^—Cu1—N1—C14	−80.29 (18)

Symmetry codes: (i) −*x*+2, −*y*+1, −*z*+2.

### Hydrogen-bond geometry (Å, °)

**Table:**

*D*—H···*A*	*D*—H	H···*A*	*D*···*A*	*D*—H···*A*
O5—H5···O2	0.84	1.67	2.461 (2)	156
O1—H1B···O6^ii^	0.83	1.93	2.763 (3)	173
O1—H1A···O4^iii^	0.83	1.89	2.706 (3)	167

Symmetry codes: (ii) −*x*+1, −*y*+1, −*z*+2; (iii) −*x*+2, −*y*+1, −*z*+1.
